# Genomic Instability and DNA Damage Responses in Progeria Arising from Defective Maturation of Prelamin A

**DOI:** 10.18632/aging.100012

**Published:** 2009-01-16

**Authors:** Phillip R. Musich, Yue Zou

**Affiliations:** Department of Biochemistry & Molecular Biology, Quillen College of Medicine, East Tennessee State University, Johnson City, TN 37614-0581, USA

**Keywords:** Lamin A, Hutchinson-Gilford progeria syndrome, premature aging, Genome instability, DNA damage responses, XPA, DNA double strand breaks, DNA repair

## Abstract

Progeria syndromes have in common a premature aging phenotype and increased genome instability.  The susceptibility to DNA damage arises from a compromised repair system, either in the repair proteins themselves or in the DNA damage response pathways.  The most severe progerias stem from mutations affecting lamin A production, a filamentous protein of the nuclear lamina. Hutchinson-Gilford progeria syndrome (HGPS) patients are heterozygous for a *LMNA* gene mutation while Restrictive Dermopathy (RD) individuals have a homozygous deficiency in the processing protease Zmpste24.  These mutations generate the mutant lamin A proteins progerin and FC-lamina A, respectively, which cause nuclear deformations and chromatin perturbations. Genome instability is observed even though genome maintenance and repair genes appear normal.  The unresolved question is what features of the DNA damage response pathways are deficient in HGPS and RD cells.  Here we review and discuss recent findings which resolve some mechanistic details of how the accumulation of progerin/FC-lamin A proteins may disrupt DNA damage response pathways in HGPS and RD cells.  As the mutant lamin proteins accumulate they sequester replication and repair factors, leading to stalled replication forks which collapse into DNA double-strand beaks (DSBs).  In a reaction unique to HGPS and RD cells these accessible DSB termini bind *Xeroderma pigmentosum* group A (XPA) protein which excludes normal binding by DNA DSB repair proteins.  The bound XPA also signals activation of ATM and ATR, arresting cell cycle progression, leading to arrested growth.  In addition, the effective sequestration of XPA at these DSB damage sites makes HGPS and RD cells more sensitive to ultraviolet light and other mutagens normally repaired by the nucleotide excision repair pathway of which XPA is a necessary and specific component.

## Introduction

The aging process represents progressive changes in a cell or an organism which culminate in death due to accumulated defects in function leading to system failure [1].  These defects result in part from accumulated damage to DNA.  Such damage may result from environmental insults such as ultraviolet (UV) and ionizing radiation, exogenous chemical and biological genotoxins, as well as endogenous mutagens (*e.g.*, reactive oxygen intermediates).  The accumulated changes lead to deficiencies in enzymes involved in necessary metabolic and maintenance processes, over time causing an escalating loss of function with an inability to maintain replicative fidelity of the genome [2-4].  Thus, organisms with mutations to genes directly involved in basic genome structure, maintenance and replicative fidelity would understandably have an accelerated aging phenotype and/or shortened life spans.

Individuals with a progeroid syndrome have a premature aging phenotype and, depending on the specific mutations involved, the effects on lifespan may range from moderate to severe.  Examples include Werner syndrome (WS), Bloom syndrome (BLM), Cockayne syndrome (CS), ataxia-telangiectasia (AT), Hutchinson-Gilford progeria syndrome (HGPS), and restrictive dermopathy (RD).  They arise from mutations in one or several genes involved in DNA metabolism or in its regulation.  Accelerated aging also may result from partial genome imbalances as seen in the chromosomal disorders of Down, Klinefelter and Turner syndromes.

WS or BLM arise from mutations in the *WRN* or *BLM* genes which encode RecQ DNA helicase proteins [5-7] while CS stems from mutations to the E xcision R epair C ross-C omplementing group 6 or 8 proteins (ERCC-6 or -8, also called CSB or CSA, respectively) [8].  Mutations to the *ATM* (a taxia-t elangiectasia m utated) gene cause AT; *ATM* encodes a phosphatidylinositol-3-kinase involved in the cell cycle checkpoint signaling pathway for detection of DNA damage and its subsequent repair [9,10]. Thus, the WRN, BLM, ERCC6/8 and ATM proteins are involved directly in DNA repair processes and their mutations cause elevated levels of genome instability, premature aging phenotypes and for ERCC8 and ATM cancer susceptibilities. Interestingly, HGPS and RD are laminopathy-based diseases; they arise not from mutated DNA metabolism genes but from mutations causing altered processing/maturation of lamin A, an intermediate-filament protein component of the nuclear lamina [6,11-16]. Nevertheless, HGPS and RD are the most severe forms of progeria; HGPS individuals have an average life span of 13.5 years while RD individuals suffer perinatal death [13,15,17]. While lamin A is not involved directly in DNA metabolism, particularly DNA repair and damage responses, DNA double-strand breaks (DSBs) are found to accumulate in HGPS and RD cells [18-20]. Similar DSB accumulation also appears to happen in physiological aging for healthy individuals who have intact DNA metabolism genes [21].  Thus, an interesting question concerns how altered lamin A proteins cause disruption of the normal organization of the nuclear genome and how such spatial disruptions cause deficiencies in DNA repair processes even though DNA repair or metabolism genes are not defective.  This review will consider the epigenetic effects of lamin A abnormalities and their perturbation of DNA damage recognition and its repair, leading to genome instability in HGPS and RD patients.

### Laminopathies in Hutchinson-Gilford progeria syndrome & restrictive dermopathy

**Figure 1.**
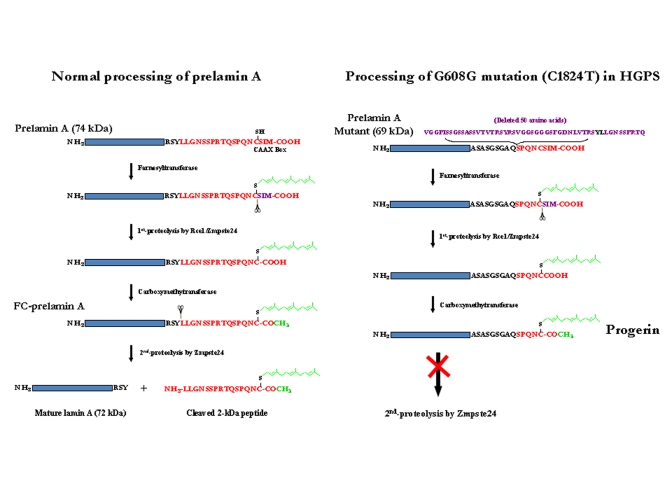
Maturation of lamin A and formation of progerin or LA∆50. **(A)** Normal processing of prelamin A. **(B)** Processing of G608G mutation (C1824T) in HGPS cells. Underline LY (in black) in the deleted 50 AAs: Zmpste24 cleavage site

The lamins are filamentous protein components of the nuclear lamina and, to a lesser extent, they form foci within the nucleoplasm in performing dynamic structural roles in the nucleus [22-24]. Lamin proteins also interact directly with histone H2A [25].   There are four major lamin proteins (A-type and B-type) in humans.  Lamins A and C (A-type) derive from alternative mRNA splicing products of the *LMNA* gene; exons 1-10 encode the N-terminal 566 amino acids of lamins A and C; however, exons 11 and 12 are unique to lamin A mRNA and code for an additional 98-amino acid C-terminal region which contains functionally important post-translational modification sites.  Lamin B1 and B2 (B-type) are encoded by *LMNB1* and *LMNB2* genes and are expressed throughout development and in adult cells.  In contrast, *LMNA* expression occurs in differentiated cell types. Lamins A and B differ from lamin C in that they are post-translationally modified in their C-terminal regions (Figure 1). The lamin B proteins retained the added farnesyl and carboxy methyl groups which are critical for their nuclear function [26].  In contrast, these prosthetic groups are removed by proteolytic cleavage in the final step of lamin A maturation processing (Figure 1).  Genetic disruptions of this final proteolytic step form the basis for HGPS and RD [15,23,27].

Prelamin A is the translation product of the mature *LMNA* mRNA in normal individuals.  This 664-amino-acid protein is post-translationally processed into lamin A by two transfer reactions and two proteolytic cleavages (Figure 1).  A farnesyl transferase specifically directs the transfer of the hydrophobic 15-carbon chain from farnesyl pyrophosphate to the cysteine at the C-terminal CAAX motif of prelamin A.  The terminal tripeptide is then proteolytically removed by either Rce-1 (Ras converting enzyme-1) or the zinc metallo-proteinase Zmpste24 (also known as FACE-1).  The terminal cysteine then is carboxy-methylated.  Prelamin B is similarly post-translationally modified to this stage.  For prelamin A the 15-amino acid C-terminal peptide containing the two modifications then is removed by a 2^nd^ Zmpste24 cleavage to generate mature lamin A [28].

The HGPS and RD laminopathies arise from deficiencies in these post-translational modifications of prelamin A.  All Zmpste24  enzymatic  activity is lost in individuals with RD (*Zmpste24^-/-^*); the farnesylated and carboxy-methylated prelamin A (**FC-prelamin A**) is toxic, especially with the absence of normal lamin A, causing perinatal death [29,30].  HGPS individuals are heterozygous for a mutation within the *LMNA* gene itself.  The dominant mutation is a CàT base substitution at position 1824 within exon 11.  Although there is no amino acid change (G608G) a cryptic splice donor site is activated within exon 11. Sporadic use of this cryptic site in splicing of *LMNA* pre-mRNA removes an additional 150 base-pair sequence, causing a 50-amino acid deletion (Figure 1) within the prelamin A protein though mature lamin A is still largely produced.  The missing region includes the second Zmpste24 cleavage site (Figure 1).  Thus, a slightly smaller farnesylated and carboxy-methylated mutant prelamin A protein (termed **progerin** or LAΔ50) forms and accumulates though at a much slower rate than for FC-prelamin A formed with the homozygous Zmpste24 mutation in RD.  While the farnesyl and carboxy-methyl moieties are necessary for lamin B functions their persistence in progerin and FC-lamin A causes multiple abnormalities in nuclear structure and function [11,16,20,23,27,31,32].  The  hydrophobic  farnesyl chain gives progerin a greater affinity for the inner nuclear membrane (INM), redistributing progerin away from nucleoplasmic foci.   This association with the INM also deforms the membrane.  During interphase, the dysmorphic nuclei are lobulated, the nuclear lamina thickens, and there is a loss of heterochromatin and nucleoplasmic lamin A foci.  The nucleoplasmic foci normally contain the replicative proteins PCNA and polymerase δ and appear to be critical for ordered initiation of genome replication in early S-phase [32,33]. Functionally, histone modification and gene expression patterns change [8,34], and DNA damage increases with a loss of DNA repair efficiency [12,18].  Cell division also is modified during nuclear envelope dissolution and reassembly. During mitosis progerin plus normal lamin A mis-localize into insoluble cytoplasmic aggregates and membranes, delaying their return to the INM and lamina of the reformed nucleus.  This causes spatial and functional disruption of interphase G_1_ chromatin and may lead to formation of bi-nucleate cells [35,36].  These structural, spatial and DNA damage/repair changes lead to increased genome instability and cytotoxicity as progerin protein accumulates in aging HGPS cells [11,23].

**Figure 2.**
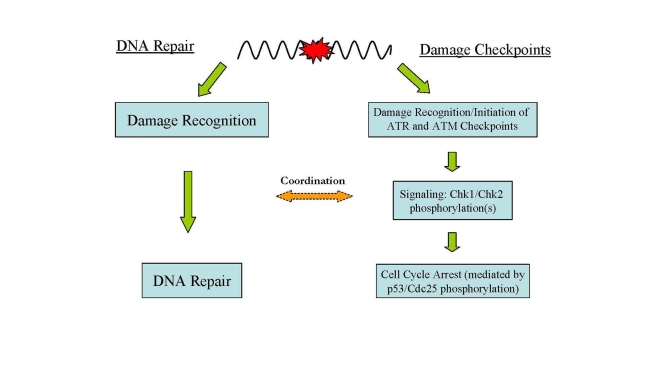
Major DNA damage responses in human cells. In response to DNA damage, two major cellular pathways, DNA damage checkpoints and DNA repair, are activated for maintaining genome integrity and stability.

### DNA damage and accumulation in HGPS and RD cells

It is generally believed that cellular DNA damage accumulation is a hallmark step leading to premature aging and the aging phenotypes featured with genome instability. Indeed, like other types of progeroid cells, HGPS and RD cells accumulate DNA damage, in particular DSBs, with continued passage in culture [12,18,19], indicating that DNA repair activity is impaired in these cells. The DSB accumulation causes genome instability, eventually leading to cellular senescence. However, unlike most types of progeria, the DNA damage accumulation in HGPS and RD is not caused by genetic deficiency in DNA repair pathways, making the laminopathy-based diseases a unique type of progeria in terms of the cause of genome instability and DNA repair dysfunction. Some insights into the molecular mechanisms responsible for DSB accumulation in HGPS and RD cells recently have been revealed and are discussed in following sections.

The laminopathy-based progeroid cells also were found to be sensitive to various DNA damaging agents. In particular, Zmpste24^-/-^ mouse embryonic fibroblasts (MEFs) are extremely sensitive to DSB inducers such as camptothecin (CPT) and etoposide [12], which is consistent with the observation of DSB accumulation in aging HGPS  and  RD patient cells.  Interestingly,  however, MEFs are also hypersensitive to UV irradiation which typically induces bulky DNA adducts exclusively removed by the nucleotide excision repair pathway (NER) [12]. In addition, MEFs are sensitive to mitomycin C, a carcinogen inducing interstrand crosslinks in DNA. However, MEFs show very limited sensitivity to the alkylating agent methyl methane-sulfonate (MMS) [12]. These cytotoxicity phenotypes reflect the deficiency in maintaining genome stability in the Zmpste-24 deficient mouse cells.

### DNA damage response signaling in HGPS and RD

HGPS and RD cells in culture exhibit limited growth potential relative to BJ normal human primary fibroblast cells.  Young HGPS and RD cells grow quite well but the cells senesce quickly relative to normal fibroblasts and growth stops, much sooner for RD than HGPS [18].  As the growth rate slows the frequency of dysmorphic nuclei increases as does the number with γ-H2AX (a marker of DNA DSBs) foci detected by immunofluorescence microscopy [11,19,37]. H2AX is a variant of histone H2A and represents a minor component of that histone in cell nuclei [38].  Histone H2AX is phosphorylated to γ-H2AX in response to DSBs in interphase cells *via* ATM signaling [39,40].  Thus, γ-H2AX has been used in immunomicroscopy to cytologically mark nuclear sites of DNA DSBs and is employed biochemically to isolate chromatin fragments containing DSBs using the Ch romatin I mmuno-P recipitation (ChIP) procedure [19].  A combination of culture ‘aging' and the specific tracking approaches of immunofluorescence microscopy, the ChIP assay and Western blotting now allow mechanistic questions to be asked concerning the deficiencies in DNA damage recognition and repair in aging progeroid cells.

DNA damage in cells evokes a checkpoint response which moderates cell cycle progression for repair of the damage [41] (Figure 2).  The first part of this process is recognition of the DNA damage and initiation of the damage response which includes activation of cell cycle checkpoints and the phosphorylation of H2AX.  The response begins with the activation of ATM and ATR (ATM- and Rad3-related) which play central roles in DNA damage checkpoints.  ATR is activated by a wide spectrum of DNA damages inducing replication stress while ATM is activated primarily by DNA DSBs [9,42,43]. Signal-transducing kinases Chk1 and Chk2 are then phosphorylated by activated ATM and ATR leading to a cascade of further down-stream activating signals (*i.e.*, phosphorylation of p53) *via* the kinase activities of Chk1 and Chk2 [41,43].

Culture-aged HGPS and RD cells contain accumulated DNA damage and compromised genome integrity.  Liu et al. examined these cells to determine if the damage checkpoint pathways were persistently activated [18].  They found that aged HGPS and RD cells contained higher levels of γ-H2AX than did normal BJ fibroblasts indicating more frequent DNA DSBs.  The progeroid cells also exhibited high levels of phosphorylated Chk1 and Chk2 due to ATM and ATR activation.  Phospho-rylated p53 is a downstream product of Chk1 and Chk2 activation and it also was increased significantly in the HGPS and RD cells. These findings demonstrate that ATR and ATM checkpoint pathways were persistently activated by the damaged DNA in the progeroid cells. While ATM and ATR were diffusely distributed in the nuclei of BJ cells, they clustered into distinct foci in nuclei of the HGPS and RD cells [18].  These foci were identical to those observed in BJ cells treated with UV irradiation (for ATR) or CPT (for ATM) [12].

Liu et al. also determined biochemically whether ATM and ATR activities were responsible for the reduced replicative capacity of HGPS cells.  Caffeine inhibits both ATM and ATR, and caffeine-treated HGPS cells demonstrated a significant restoration of replicative activity.  Knockdown of ATM and ATR protein levels by siRNA silencing also restored significant replicative activity [18].  Thus, the decreased cell cycling observed in aged progeroid cells is one response to the accumulated DNA damage which is mediated by ATM and ATR checkpoint pathways.

Are the activation and sub-nuclear clustering of ATM and ATR in progeroid cells directly related to the accumulated progerin protein?  This question was addressed by investigating the effects of progerin expression in normal cells and, alternatively, the inhibition of the prelamin A processing in progeroid cells [18].  It was observed that HeLa cells transfected with a progerin-expressing plasmid exhibited ATR nuclear foci formation, demonstrating that foci formation is progerin-dependent.  Inhibition of the prenylation of G608G mutant prelamin A with the farnesyl transferase inhibitor L-744832 restored normal nuclear shape. Interestingly, however, the levels of γ-H2AX and phosphorylated Chk1 and Chk2 in HGPS cells were not reduced.  Thus, reversal of dysmorphic nuclei formation has no effect on cell cycle checkpoint activation from existing DNA DSBs.

###  Deficiencies in DNA damage recognition and repair in HGPS and RD

Genome instability can arise from multiple causes; one of the most obvious being an increased sensitivity to DNA damage due to genetic or epigenetic deficiencies in DNA repair.  The persistent activation of ATM/ATR checkpoint pathways in HGPS and RD reflects a delay in DNA repair efficiency in these cells [18].  The DSB accumulation in these cells is particularly puzzling since HGPS and RD cells are genetically defective in prelamin A and related processing pathways rather than in DNA repair proteins.

It is expected that multiple DSB repair proteins would be recruited to the DNA damage sites for repair as part of the damage response.  Surprisingly, such was not the case.  Employing immunofluorescence tracking of γ-H2AX foci and neutral single-cell electrophoretic (comet) assays to measure DNA DSBs Zou's group observed a significant parallel increase in nuclear γ-H2AX foci and DSB frequency in HGPS cells relative to BJ fibroblasts.  Cellular progerin levels exhibited similar increases in the aged progeroid cells [19].  Although elements of  the damage response system (*i.e.*, ATR, ATM, Chk1, Chk2 and p53) were activated [18], immunofluorescence studies indicated that nuclear foci of Rad50 or Rad51 did not colocalize with the γ-H2AX foci in HGPS and RD cells [19].  This was unexpected since Rad50 (part of the MRN complex of Mre11/Rad50/Nbs1) and Rad51 are components critical for repair of DNA DSBs [41,44-46] and for the restart of stalled replication forks [47].  In contrast, DSBs induced in normal BJ cells by CPT showed colocalization of γ-H2AX with Rad50 or Rad51 foci.  The failed recruitment of repair factors to the laminopathy-induced DSBs made the DNA damage unrepairable in HGPS and RD cells [19]. Impaired recruitment to DSB foci of Rad51 and 53BP1 (p53-binding protein 1) also was observed in bone marrow cells of Zmpste24^-/-^mice and in HGPS cells treated with γ-irradiation [12]. These data raise the question of why these repair proteins were not recruited to the DSB sites.

*Xeroderma pigmentosum* group A (XPA) protein is a specific and essential factor for NER but is not involved in the repair of DSBs [41].  The role of XPA in NER is believed to include DNA damage recogni-tion/verification, NER nuclease recruiting, and stabilization of repair intermediates [41,48-51]. NER does not process DSBs nor does it introduced DSB intermediates during the repair process.  Surprisingly, XPA colocalized with the γ-H2AX sites of DSBs in HGPS and RD cells [19].  XPC is the major DNA damage recognition protein in NER [41] but did not exhibit nuclear foci in HGPS and RD cells indicating that the colocalization of XPA and γ-H2AX was specific and not related to NER [19]. Furthermore, in HGPS and RD cells treated with CPT (a DSB-inducer) XPA did not colocalize to these CPT-induced DSBs though it still colocalized to the endogenous laminopathy-induced DSB foci.  Also, the CPT-induced foci were repaired in HGPS and RD cells, though at a slower rate than in the BJ cells. The latter result demonstrates that the DSB repair system *per se* in HGPS and RD cells is functional, and, also that the XPA behaves normally in not binding to genotoxin-induced DSBs.

How does the binding of XPA to laminopathy-generated DSBs relate to the lack of Rad50 and Rad51 binding?  Is the XPA association with the DSBs sufficient to exclude these proteins?  Zou's group employed the ChIP assay and siRNA knockdown of XPA to resolve these questions.  XPA was found in the γ-H2AX-associated chromatin fragments from HGPS cells but not from normal BJ cells, even when DSBs were induced in the latter by CPT [19].  Nuclease treatment of the chromatin before immunoprecipitation released the XPA from the γ-H2AX chromatin complex.  Thus, DNA mediates the association of XPA and γ-H2AX-marked chromatin containing DNA DSBs.

If this XPA association with DSBs in progeroid chromatin is sufficient to exclude Rad50 and Rad51, this exclusion should be reversible with XPA depletion by knockdown with RNAi.  Lui et al*.* observed that XPA depletion partially restored the recruitment of Rad50, Rad51 and Ku70 to γ-H2AX chromatin containing DNA DSBs [19,52].  This confirms that the binding of XPA to laminopathy-induced DSBs in HGPS and RD cells disrupted recruitment of factors normally involved in their repair. This is further supported by their finding that XPA depletion significantly reduced the level of DSBs in HGPS cells but had no effect on CPT-induced DSB level in BJ cells. Thus, XPA binding to DNA DSBs in progeroid cells may explain the absence of appropriate repair proteins at these sites and the genome instability observed in these cells due to failure to execute DNA repair.

Bomgarden et al. found that of the multiple NER factors XPA specifically was needed for ATR signaling of DNA damage during S-phase and that XPA knockdown compromised the normal response to UV damage [53].  This is consistent with the role of XPA in verifying the presence of bulky lesions in NER [54-56].  The proportion of HGPS cells in S-phase increases with cell age as does the level of accumulated DSBs. Thus, it would be interesting to see if the localization of XPA to these damage sites is required for activation of ATM and ATR checkpoint pathways in HGPS and RD cells [18].

**Figure 3.**
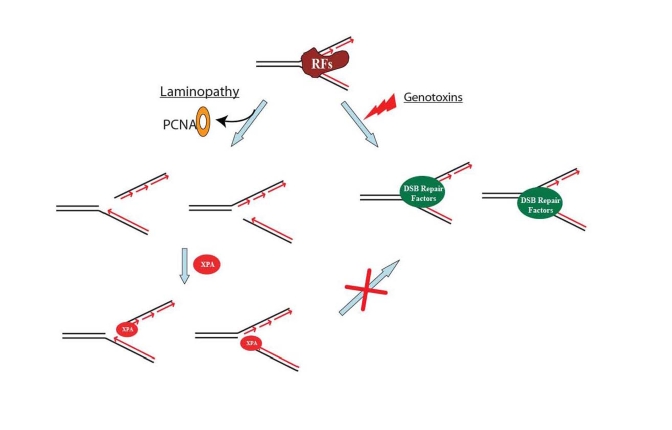
A proposed model showing that DNA double-strand break repair activity is impaired in HGPS and RD cells. Unlike the replication fork collapse induced by genotoxins, laminopathy-induced replication fork collapse may be characterized with a possible loss of PCNA at replication forks. The subsequent possible binding of XPA to the "naked" replication forks with DNA double-strand breaks (DSBs) blocks the access of DSB repair proteins to the damage sites. RFs stands for replication factors.

Lamin A and C proteins form nucleoplasmic foci which organize proteins for initiation of replication in early S-phase, including the colocalization of PCNA [32]. Microinjection of an N-terminal mutant lamin A protein (ΔNLA) disrupts the nuclear lamina organization in mammalian cells and causes a redistribution of the replication elongation proteins PCNA and RFC [57,58].  The absence of PCNA at replication centers due to its sequestration in ΔNLA-lamins aggregates in a dominant-negative manner may lead to stalled replication forks; collapse of the replication forks may result in DSBs [59].  Shumaker et al. also observed that the Ig-fold domain of all lamin proteins bound directly to PCNA and that excess amounts of the Ig-fold domain sequestered the PCNA and inhibited DNA replication [60].  The Ig-fold domain occurs just before the CAAX-box which is modified in the laminopathies (see Figure 1).  Progerin and FC-prelamin A, the mutant forms of lamin A in HGPS and RD cells, respectively (Figure 1; [6,11-16]), are known to disrupt normal nuclear structure including the perinucleolar lamin A/C granules containing the replicative proteins PCNA and polymerase δ [33].  If these progeroid proteins generate a redistribution of PCNA and/or RFC, they also would cause replication fork stalling followed by DNA DSB formation.  During this process, the replication fork and its damage intermediates, now PCNA- and RFC-deficient, may become accessible for XPA binding.  The bound XPA then blocks association of DSBs with the repair proteins Rad50, Rad51 and 53BP1 [12,19] (Figure 3).  PCNA forms discrete nuclear foci in early-passage HGPS cells [61] when no XPA foci were seen.  However, PCNA foci were not seen in late-passage cells (unpublished data) when there is an increase in XPA foci colocalizing with γ-H2AX and in DNA DSBs [19].

Why does XPA colocalize with the laminopathy-induced DSBs marked by γ-H2AX in aging progeroid cells?  Stalled replication forks may result in S-phase arrest via persistent ATM/ATR activation [18,53].  DSBs can be generated at stalled forks [59,62-64] that contain strand termini of double-stranded/single-stranded DNA (ds-ssDNA) junctions, mostly from Okazaki fragments.  A recent study indicated that XPA exhibits an affinity for these ds-ssDNA junctions even higher than its affinity for the DNA damage processed by NER [51].  In HGPS cells, the possible sequestration of PCNA at functioning replication forks and in progerin aggregates may leave the strand termini of ds-ssDNA junctions unprotected, allowing access to XPA for binding (Figure 3).   Thus, the amount of progerin increases with age in progeroid cells, as does the number of nuclear γ-H2AX foci and measurable DSBs as well as XPA foci [19].  In addition, the unexpected translocation of XPA to the DSB sites in progeroid cells may trap this NER protein at the collapsed replication forks, which subsequently may silence NER activity for repair of bulky DNA adducts such as the photoproducts induced by UV irradiation.  This may explain the observed hypersensitivity of progeroid cells to UV damage in addition to DSB damage [12].

### Therapeutic strategies for treatment of HPGS

Farnesyl transferase inhibitors (FTIs) have been applied to progeroid cells and to Zmpste24^-/-^ mice to block the prenylation reaction since it is believed that a major phenotype-inducing element of progerin and FC-prelamin A is the farnesyl moiety [14,29].  FTI treatment did reduce farnesylated forms of progerin and FC-prelamin A and correct the nuclear dysmorphology [65,66]. However, FTI treatment of progeroid cells did not reduce the frequency of DNA DSBs nor the levels γ-H2AX protein and its nuclei foci [12,19,52]. Consistently these proteins were prenylated instead by geranylgeranyl addition and some of the laminopathy conditions persisted [67,68].  The prenyl groups are derived from the cholesterol biosynthetic pathway; statins and amino-bisphosphonates are common drugs for treatment of hypercholesterolemia [29].  These drugs also appear more effective than FTIs in reducing phenotypic markers of laminopathy in model mice and cellular (HGPS, RD) assays [29,67,68]. It will be of interest to determine whether the statin/amino-bisphosphonate drug combination will be more effective in reducing aberrant nuclear morphology and genome instability phenotypes.

### HGPS and normal aging

Great interest in understanding HGPS has been promoted by recent findings that linked normal aging to the laminopathy disease. The connection is supported by several lines of evidence and observation. First, the same mechanism responsible for HGPS is also active in normal aging cells [21]. Cells from healthy individuals also express low levels of progerin from sporadic use of the cryptic splice site [21], resulting in similar phenotypes. For instance, the level of γ-H2AX increases with an individual's age in tissue samples and with time in culture for primary cell explants [21,37,39], which is concomitant with a parallel increase in laminopathy-induced DNA damage and the pathological changes in nuclear morphology and chromatin structures. Secondly, like in HGPS, DNA damage accumulation in healthy aging cells is not caused by a genetic deficiency in DNA repair. It is quite likely that the same sporadic abnormal splicing of prelamin A mRNA is responsible for the genome instability in both HGPS and normal aging. Finally, like in HPGS, DSBs formed in normal human aging also are unrepairable although genotoxin-induced DSBs in the same cells can be efficiently repaired [2].  All these mechanistic similarities strongly support the use of HGPS or related laminopathies as an excellent model for the study of normal human aging.

## Conclusion

Genome instability caused by cellular accumulation of DNA damage, particularly DNA double-strand breaks, is a common cause of systemic aging and premature aging [2-4]. However, how and why DNA damage accumulates in healthy aging cells and laminopathy-based premature aging cells are far from clear. The questions are particularly intriguing and challenging as these cells appear to contain genetically intact DNA repair machineries and are expected to be able to maintain genome integrity. In this regard, recent studies have shed new light on the molecular basis of genome instability and DNA damage responses in these cells.  Findings from these studies indicate that DSBs accumulate in HGPS and Zmpste24^-/-^cells as well as normal aging cells which also express low levels of progerin. The endogenously induced DNA damage in these cells is unrepairable and concurrent with aberrant nuclear morphology. In HGPS and RD cells, however, the accumulated damage can not be reversed by treatment with FTIs though the treatment restores the normal nuclear morphology of the cells. In response to the accumulated DNA damage, ATM and ATR checkpoints are highly and persistently activated in these progeroid cells, leading to accelerated replicative arrest. Importantly, the fact that DNA damage is unrepairable is at least in part due to a "murder-suicide" action mediated by wild-type NER protein XPA which is unexpectedly trapped to DSB sites. The action not only blocks the access of DSBs to DSB repair factors but also abolishes NER to which XPA belongs. This mechanism also represents the first known case in which a protein from one DNA repair pathway disrupts another DNA repair pathway. Due to the common involvement of progerin in both HGPS and normal aging, it will be of great interest to see if the same mechanism is also true in normal aging. In addition, outstanding questions as to what is the cause for XPA mislocalization to the DSB sites and what is the epigenetic role of progerin in this process remain to be addressed in the future.

## References

[1] Kirkwood TB (2005). Understanding the odd science of aging. Cell.

[2] Sedelnikova OA, Horikawa I, Zimonjic DB, Popescu NC, Bonner WM, Barrett JC (2004). Senescing human cells and ageing mice accumulate DNA lesions with unrepairable double-strand breaks. Nat Cell Biol.

[3] Gorbunova V, Seluanov A (2005). Making ends meet in old age: DSB repair and aging. Mech Ageing Dev.

[4] Lombard DB, Chua KF, Mostoslavsky R, Franco S, Gostissa M, Alt FW (2005). DNA repair, genome stability, and aging. Cell.

[5] Ellis NA, German J (1996). Molecular genetics of Bloom's syndrome. Hum Mol Genet.

[6] Kudlow BA, Kennedy BK, Monnat RJ Jr (2007). Werner and Hutchinson-Gilford progeria syndromes: mechanistic basis of human progeroid diseases. Nat Rev Mol Cell Biol.

[7] Martin GM (2005). Genetic modulation of senescent phenotypes in Homo sapiens. Cell.

[8] Kyng KJ, Bohr VA (2005). Gene expression and DNA repair in progeroid syndromes and human aging. Ageing Res Rev.

[9] Shiloh Y (2001). ATM and ATR: networking cellular responses to DNA damage. Curr Opin Genet Dev.

[10] Cimprich KA, Cortez D (2008). ATR: an essential regulator of genome integrity. Nat Rev Mol Cell Biol.

[11] Goldman RD, Shumaker DK, Erdos MR, Eriksson M, Goldman AE, Gordon LB, Gruenbaum Y, Khuon S, Mendez M, Varga R, Collins FS (2004). Accumulation of mutant lamin A causes progressive changes in nuclear architecture in Hutchinson-Gilford progeria syndrome. Proc Natl Acad Sci U S A.

[12] Liu B, Wang J, Chan KM, Tjia WM, Deng W, Guan X, Huang JD, Li KM, Chau PY, Chen DJ, Pei D, Pendas AM, Cadinanos J, Lopez-Otin C, Tse HF, Hutchison C, Chen J, Cao Y, Cheah KS, Tryggvason K, Zhou Z (2005). Genomic instability in laminopathy-based premature aging. Nat Med.

[13] Misteli T, Scaffidi P (2005). Genome instability in progeria: when repair gets old. Nat Med.

[14] Pereira S, Bourgeois P, Navarro C, Esteves-Vieira V, Cau P, De Sandre-Giovannoli A, Lévy N (2008). HGPS and related premature aging disorders: From genomic identification to the first therapeutic approaches. Mech Ageing Dev.

[15] Smith ED, Kudlow BA, Frock RL, Kennedy BK (2005). A-type nuclear lamins, progerias and other degenerative disorders. Mech Ageing Dev.

[16] Wiesel N, Mattout A, Melcer S, Melamed-Book N, Herrmann H, Medalia O, Aebi U, Gruenbaum Y (2008). Laminopathic mutations interfere with the assembly, localization, and dynamics of nuclear lamins. Proc Natl Acad Sci U S A.

[17] Scaffidi P, Gordon L, Misteli T (2005). The cell nucleus and aging: tantalizing clues and hopeful promises. PLoS Biol.

[18] Liu Y, Rusinol A, Sinensky M, Wang Y, Zou Y (2006). DNA damage responses in progeroid syndromes arise from defective maturation of prelamin A. J Cell Sci.

[19] Liu Y, Wang Y, Rusinol AE, Sinensky MS, Liu J, Shell SM, Zou Y (2008). Involvement of Xeroderma pigmentosum group A (XPA) in progeria arising from defective maturation of prelamin A. FASEB J.

[20] Scaffidi P, Misteli T (2005). Reversal of the cellular phenotype in the premature aging disease Hutchinson-Gilford progeria syndrome. Nat Med.

[21] Scaffidi P, Misteli T (2006). Lamin A-dependent nuclear defects in human aging. Science.

[22] Hutchison CJ (2002). Lamins: building blocks or regulators of gene expression. Nat Rev Mol Cell Biol.

[23] Dechat T, Pfleghaar K, Sengupta K, Shimi T, Shumaker DK, Solimando L, Goldman RD (2008). Nuclear lamins: major factors in the structural organization and function of the nucleus and chromatin. Genes Dev.

[24] Houben F, Ramaekers FC, Snoeckx LH, Broers JL (2007). Role of nuclear lamina-cytoskeleton interactions in the maintenance of cellular strength. Biochim Biophys Acta.

[25] Mattout A, Goldberg M, Tzur Y, Margalit A, Gruenbaum Y (2007). Specific and conserved sequences in D. melanogaster and C. elegans lamins and histone H2A mediate the attachment of lamins to chromosomes. J Cell Sci.

[26] Rusinol AE, Sinensky MS (2006). Farnesylated lamins, progeroid syndromes and farnesyl transferase inhibitors. J Cell Sci.

[27] Dahl KN, Scaffidi P, Islam MF, Yodh AG, Wilson KL, Misteli T (2006). Distinct structural and mechanical properties of the nuclear lamina in Hutchinson-Gilford progeria syndrome. Proc Natl Acad Sci U S A.

[28] Corrigan DP, Kuszczak D, Rusinol AE, Thewke DP, Hrycyna CA, Michaelis S, Sinensky MS (2005). Prelamin A endoproteolytic processing in vitro by recombinant Zmpste24. Biochem J.

[29] Navarro CL, Cau P, Levy N (2006). Molecular bases of progeroid syndromes. Hum Mol Genet.

[30] Navarro CL, De Sandre-Giovannoli A, Bernard R, Boccaccio I, Boyer A, Genevieve D, Hadj-Rabia S, Gaudy-Marqueste C, Smitt HS, Vabres P, Faivre L, Verloes A, Van Essen T, Flori E, Hennekam R, Beemer FA, Laurent N, Le Merrer M, Cau P, Levy N (2004). Lamin A and ZMPSTE24 (FACE-1) defects cause nuclear disorganization and identify restrictive dermopathy as a lethal neonatal laminopathy. Hum Mol Genet.

[31] Houben F, Willems CH, Declercq IL, Hochstenbach K, Kamps MA, Snoeckx LH, Ramaekers FC, Broers JL (2008). Disturbed nuclear orientation and cellular migration in A-type lamin deficient cells. Biochim Biophys Acta.

[32] Kennedy BK, Barbie DA, Classon M, Dyson N, Harlow E (2000). Nuclear organization of DNA replication in primary mammalian cells. Genes Dev.

[33] Barbie DA, Kudlow BA, Frock R, Zhao J, Johnson BR, Dyson N, Harlow E, Kennedy BK (2004). Nuclear reorganization of mammalian DNA synthesis prior to cell cycle exit. Mol Cell Biol.

[34] Shumaker DK, Dechat T, Kohlmaier A, Adam SA, Bozovsky MR, Erdos MR, Eriksson M, Goldman AE, Khuon S, Collins FS, Jenuwein T, Goldman RD (2006). Mutant nuclear lamin A leads to progressive alterations of epigenetic control in premature aging. Proc Natl Acad Sci U S A.

[35] Dechat T, Shimi T, Adam SA, Rusinol AE, Andres DA, Spielmann HP, Sinensky MS, Goldman RD (2007). Alterations in mitosis and cell cycle progression caused by a mutant lamin A known to accelerate human aging. Proc Natl Acad Sci U S A.

[36] Cao K, Capell BC, Erdos MR, Djabali K, Collins FS (2007). A lamin A protein isoform overexpressed in Hutchinson-Gilford progeria syndrome interferes with mitosis in progeria and normal cells. Proc Natl Acad Sci U S A.

[37] Sedelnikova OA, Horikawa I, Redon C, Nakamura A, Zimonjic DB, Popescu NC, Bonner WM (2008). Delayed kinetics of DNA double-strand break processing in normal and pathological aging. Aging Cell.

[38] Redon C, Pilch D, Rogakou E, Sedelnikova O, Newrock K, Bonner W (2002). Histone H2A variants H2AX and H2AZ. Curr Opin Genet Dev.

[39] Kinner A, Wu W, Staudt C, Iliakis G (2008). {gamma}-H2AX in recognition and signaling of DNA double-strand breaks in the context of chromatin. Nucl Acids Res.

[40] Rogakou EP, Boon C, Redon C, Bonner WM (1999). Megabase chromatin domains involved in DNA double-strand breaks in vivo. J Cell Biol.

[41] Sancar A, Lindsey-Boltz LA, Unsal-Kacmaz K, Linn S (2004). Molecular mechanisms of mammalian DNA repair and the DNA damage checkpoints. Annu Rev Biochem.

[42] Abraham RT (2001). Cell cycle checkpoint signaling through the ATM and ATR kinases. Genes Dev.

[43] Li L, Zou L (2005). Sensing, signaling, and responding to DNA damage: Organization of the checkpoint pathways in mammalian cells. J Cell Biochem.

[44] Lee JH, Paull TT (2004). Direct activation of the ATM protein kinase by the Mre11/Rad50/Nbs1 complex. Science.

[45] Lee JH, Paull TT (2005). ATM activation by DNA double-strand breaks through the Mre11-Rad50-Nbs1 complex. Science.

[46] Paull TT, Lee JH (2005). The Mre11/Rad50/Nbs1 complex and its role as a DNA double-strand break sensor for ATM. Cell Cycle.

[47] Trenz K, Smith E, Smith S, Costanzo V (2006). ATM and ATR promote Mre11 dependent restart of collapsed replication forks and prevent accumulation of DNA breaks. EMBO J.

[48] Guzder SN, Sommers CH, Prakash L, Prakash S (2006). Complex formation with damage recognition protein Rad14 is essential for Saccharomyces cerevisiae Rad1-Rad10 nuclease to perform its function in nucleotide excision repair in vivo. Mol Cell Biol.

[49] Liu Y, Liu Y, Yang Z, Utzat C, Wang G, Basu AK, Zou Y (2005). Cooperative interaction of human XPA stabilizes and enhances specific binding of XPA to DNA damage. Biochemistry.

[50] Wu X, Shell SM, Yang Z, Zou Y (2006). Phosphorylation of nucleotide excision repair factor Xeroderma pigmentosum group A by ataxia telangiectasia mutated and Rad3-related-dependent checkpoint pathway promotes cell survival in response to UV irradiation. Cancer Res.

[51] Yang Z, Roginskaya M, Colis LC, Basu AK, Shell SM, Liu Y, Musich PR, Harris CM, Harris TM, Zou Y (2006). Specific and efficient binding of Xeroderma pigmentosum complementation group A to double-strand/single-strand DNA junctions with 3'- and/or 5'-ssDNA branches. Biochemistry.

[52] Liu Y (2006). Ph. D. Dissertation:
                                        1, Structural and functional studies of human replication protein A; 2, DNA
                                        damage responses and DNA repair defects in laminopathy-based premature aging. in Biochemistry & Molecular Biology.

[53] Bomgarden RD, Lupardus PJ, Soni DV, Yee MC, Ford JM, Cimprich KA (2006). Opposing effects of the UV lesion repair protein XPA and UV bypass polymerase eta on ATR checkpoint signaling. EMBO J.

[54] Riedl T, Hanaoka F, Egly JM (2003). The comings and goings of nucleotide excision repair factors on damaged DNA. EMBO J.

[55] Sugasawa K, Ng JM, Masutani C, Iwai S, van der Spek PJ, Eker AP, Hanaoka F, Bootsma D, Hoeijmakers JH (1998). Xeroderma pigmentosum group C protein complex is the initiator of global genome nucleotide excision repair. Mol Cell.

[56] Volker M, Mone MJ, Karmakar P, van Hoffen A, Schul W, Vermeulen W, Hoeijmakers JH, van Driel R, van Zeeland AA, Mullenders LH (2001). Sequential assembly of the nucleotide excision repair factors in vivo. Mol Cell.

[57] Moir RD, Spann TP, Herrmann H, Goldman RD (2000). Disruption of nuclear lamin organization blocks the elongation phase of DNA replication. J Cell Biol.

[58] Spann TP, Moir RD, Goldman AE, Stick R, Goldman RD (1997). Disruption of nuclear lamin organization alters the distribution of replication factors and inhibits DNA synthesis. J Cell Biol.

[59] Mirkin EV, Mirkin SM (2007). Replication fork stalling at natural impediments. Microbiol Mol Biol Rev.

[60] Shumaker DK, Solimando L, Sengupta K, Shimi T, Adam SA, Grunwald A, Strelkov SV, Aebi U, Cardoso MC, Goldman RD (2008). The highly conserved nuclear lamin Ig-fold binds to PCNA: its role in DNA replication. J Cell Biol.

[61] Leonhardt H, Rahn HP, Weinzierl P, Sporbert A, Cremer T, Zink D, Cardoso MC (2000). Dynamics of DNA replication factories in living cells. J Cell Biol.

[62] Cox MM (2002). The nonmutagenic repair of broken replication forks via recombination. Mutat Res.

[63] Heller RC, Marians KJ (2006). Replisome assembly and the direct restart of stalled replication forks. Nat Rev Mol Cell Biol.

[64] Liu E, Lee AY, Chiba T, Olson E, Sun P, Wu X (2007). The ATR-mediated S phase checkpoint prevents rereplication in mammalian cells when licensing control is disrupted. J Cell Biol.

[65] Glynn MW, Glover TW (2005). Incomplete processing of mutant lamin A in Hutchinson-Gilford progeria leads to nuclear abnormalities, which are reversed by farnesyltransferase inhibition. Hum Mol Genet.

[66] Young SG, Meta M, Yang SH, Fong LG (2006). Prelamin A farnesylation and progeroid syndromes. J Biol Chem.

[67] Meshorer E, Gruenbaum Y (2008). Rejuvenating premature aging. Nat Med.

[68] Varela I, Pereira S, Ugalde AP, Navarro CL, Suarez MF, Cau P, Cadinanos J, Osorio FG, Foray N, Cobo J, de Carlos F, Levy N, Freije JM, Lopez-Otin C (2008). Combined treatment with statins and aminobisphosphonates extends longevity in a mouse model of human premature aging. Nat Med.

